# Editors’ Table

**Published:** 1857-03

**Authors:** 


### Editors' Table.

QUIS CUSTODIET CUSTODES.—We are just passing through the annual season of medical graduation; the great heart of Alma Mater is contracting, and with its yearly systole will send out, *per saltum*, fifteen hundred new-fledged doctors.

The one fact that so large a number of young men are thus sent forth to the responsible duties of the medical profession, is serious past all jesting. They go forth a mighty engine of good or evil, naturally predisposed to value their profession in proportion to the difficulty experienced in procuring a foothold in it; and it becomes a weighty question for us to consider, whether or not the colleges have been conscientious and thorough in the performance of their function of license.

The journals and the speech-makers of our societies and conventions have from time to time indulged in diatribes on the faults of our collegiate system, the struggles of competition between rival schools, and what they are pleased to call the annual sale of diplomas. These charges have been so freely bandied about, that it is raising no new question to discuss them, and we propose to say a few words in defence of a system which we cherish, if not of men whom we honor. It is not our purpose to be formal or methodical, but we do not recollect to have seen the whole truth told of late. It is the fashion to lay the sins of the profession on the heads of the colleges, and fashion makes slaves of us all.

Necessarily, all faults in American medical education are chargeable to two sources.

1. Errors in the system itself: and

2. Derelictions of duty on the part of the colleges.

And, first, of the system; for although we have no law which governs our action under penalties for non-observance, we have a plan of instruction which is remarkably uniform throughout the country. In nine cases out of ten, the student enters the office of a practitioner in the spring, reads for a year and a half, attends lectures for four or five months, reads again until fall, when he again listens to lectures until the last week in February, and then receives a diploma. His early education may or may not have been good; his diligence during his term of study may or may not have been great; and everything finally hinges on his examination for a degree. The power rests in the college exclusively; if its standard is high, it matters but little about the preliminaries; a good student becomes a good doctor of medicine; while, on the other hand, a low standard of graduation must inevitably fill the ranks of the profession with inferior men.

It follows from the above, and from other facts equally well known, that the colleges are an autocracy, having in their hands the honor and true interests of the profession. It is no logical sequence from this that the system is faulty. Such a power must rest somewhere, and the fewer repositories for such a responsibility, the more readily can they be controlled.

Looking at the broad result, we have no reason for supposing that this trust is not faithfully fulfilled. We mean that in other countries, where the colleges are hemmed in by other powers, where the professorships are government offices, and the temptation to bid for large classes is removed, the evils of which it is the habit of American journalism to complain exist in spite of their more elaborate system. European education, coupled with old-world habits of thought and action, produces a limited number of splendid scholars; but it is our honest belief, that for keen, practical, useful, bedside knowledge, no part of the world has a more competent medical profession than the United States. We speak from a somewhat extensive acquaintance both in city and country.

Lying back behind all systems and schemes of government is one great influence which must not be forgotten. It was the frequent remark of a shrewd gentleman of extensive knowledge of men, that "one must not expect all the Christian virtues for eight dollars a month." There is no power of public law or public opinion that can make a good physician, except it gives him a fair remuneration for his services. Talent is in the market, and, if commerce will pay better than professional life, it will engage in commerce.

Without dwelling upon this idea, we wish merely to throw it out as suggestive of another. All projects to cheapen medical education are erroneous. We must, in considering this, disconnect our minds from European habits of thought, and look to the broad, vigorous American character as the basis of argument. The government schools of Europe are founded on aristocratic institutions, and are a part of a social system which permeates all ranks and governs all conditions in life. It would be a miracle if such a plan could be adapted to two races of people, living under such widely different influences. Even in monarchical countries, where the foundational idea of education is the same, we find wide variations in its development, which evidently depend on the national character. Thus England has its medical departments in its Universities at Cambridge and Oxford, but their existence is hardly known. Rich in endowments, surrounded by the prestige of Isis and Cam, they are feeble nonentities in the strong medical life of England. The schools of London, competitive, expensive, comparatively poor in funds, but rich in clinical advantages, the energy of an immense city, and the talent drawn to them by the great reward which success in London implies, have dwarfed and overshadowed all other means of education. So, too, in Paris, where gratuitous schools are aided by magnificent clinical advantages. The lecture-rooms of royal professors are empty, while humble private instruction draws around it crowds of students.

We have said enough to indicate our belief, that the European system is not to be desired on this side of the Atlantic. We want an American system, one adapted to the national spirit, which would refuse even the priceless blessing of education when offered as a charity. Indeed this is a proper and manly pride in a country so rich as ours, where the young man who fails can only accuse his own lack of energy or forethought.

What modifications of our present scheme of education are desirable?

Shall we link in our state governments in their control, by establishing a supervisory board having power to annul the diploma granted by the college? Such an idea has been broached, but it is only necessary to call to mind the changeful, and too frequently venal source of appointment of such a board, to see that instead of a conservative, it would be a radical movement calculated to encourage the evil it was meant to avoid. Or shall we deprive the colleges of the power to grant diplomas, giving their function to a State Board of Regents? One has only to think of the small political doctors who would be sure to receive the appointment of Regent, to be convinced that the profession, while it is too democratic for European methods, is too conservative for modern American politics.

So long as our political life is what it now is, and so long as public opinion is stronger and less easily evaded than any other force which can be brought to bear, we must trust to our present plan, or to some simple modification of it. The profession itself, speaking in its conventions, its societies, and through its press, is the only authority which can or ought to govern the colleges. There may be—doubtless, are faults, and grave ones, in the manner of granting diplomas to candidates for the doctorship; but we do not believe that these are inseparable from the system, or to be mended by any wholesale tinkering of so-called reformers. The very feature, of which loudest complaint is made — competition — is its best safeguard. We are sick of their cry of "Competition!" as if all heresy and schism were embodied in it. It is a grand, noble feature in our schools; and we hope never to see the day, when they shall be deprived of its healthy, life-giving stimulus.

We are too "intensely respectable." A school cannot flourish without advertising; and the common-sense plan is, to let the advertisement show forth the advantages claimed by the school. It is through these advertisements that competition manifests itself, and attains to its natural results,—a perpetuation of itself, and a tendency to drive schools to add to their talent, and increase their advantages. We do not assert that the use of competitive means may not be liable to great abuses; but eye hath not yet seen perfection, and we must look to consequences, before we attempt to remedy even an acknowledged evil. It is evident, that, without competition, we should have fewer schools with greater powers, that, instead of struggling to employ a high order of talent to fill their museums, and surround themselves with abundant means of clinical instruction, we should have schools of doctrine, instead of instruction, and nests of nepotism, instead of hives of labor.

Even the control of a secular board of trustees is only desirable, in the same light that a veto power is necessary in the freest government. The most intelligent board is quite as likely to fill a chair badly, as well; while the faculty can have only one motive in choosing a colleague—the best interests of the school itself. The faculty should always possess the presidential power of nomination, leaving to the senatorial trustees the function of confirmation or rejection.

But—the reader will ask—what limit should be placed upon the privileges of the colleges?

We may answer, that the most important interest of the profession at large, is in securing ample means of instruction for young men entering on the field of practice. In our opinion, looking at the character and wants of the American people, our present system does not need radical alteration; and, at the present moment, the colleges should simply be made to feel the pressure of professional approval or disapprobation, as either may seem best. Let the State societies watch the schools, and assert their prerogative of censure if they are unworthy. There is not a school in the country so strong as to outlive the deserved and formal disapprobation of the American Medical Association.

There are other matters on which we fain would touch, had we time or space to do so. Various plans have been suggested and indorsed by the National Association, looking to the more thorough qualification of the young practitioner. Among these was a recommendation to require the student to pass an examination preliminary to his entrance upon his studies. That this has not been carried out, is due to the negligence of the minor societies, which have always failed to insist upon it.

Another and more striking reform was a project to lengthen the lecture term, and bring the daily duties of the student down to an amount of instruction such as he might have time to digest. Our present six hours per diem, for sixteen weeks, is preposterous,—in violation of all laws of health, physical or mental, and should be reformed. Why no change has been wrought in this obvious evil, is past our comprehension.

It would seem, that that school, in a great city, which should announce a collegiate year, with terms and vacations of appropriate length, offering a talent fully equal to that now concentrated in the winter term, with equal clinical advantages, would at once assume a leading position. There are in the country many students of sufficient means to avail themselves of such an opportunity, and they might be concentrated. We can foresee many difficulties in this scheme, but we do not look upon them as insuperable; and let the thing be once successful at a single point, competition and professional opinion would drive every school having any claim to respectability, into a similar arrangement.

Here is a practicable, tangible plan of reformation. Let us try, and perfect our present means of education, before rushing blindly into the adoption of foreign and incongruous systems.

---

Efforts are being made this winter, in Maine and Ohio, to get the Legislatures of these respective commonwealths to pass bills legalizing the study of anatomy. That these efforts may be crowned with success, is a consummation devoutly to be wished. It was, for a long time, a disputed question, whether the protection of the law did not rather hinder than aid the procurement of anatomical material; but this has been decided by the present state of things in Massachusetts, where, to her credit be it said, was made the first experiment of placing anatomical pursuits upon a legal basis. The first effect, it is true, was anything but encouraging; but now, that the knowledge of the law has passed out of the minds of that class of individuals, who can benefit society only by allowing their carcasses to fall into the hands of the anatomist; or rather, since the people have become familiarized to the subject, material is said to be as abundant this winter, in Boston, as in any of the other Northern cities. This same first effect is stated as being experienced now in New York, an anatomical bill having been in force in that State only about a year or eighteen months; but, we feel confident that the ultimate result will be for the good of all concerned. And when—may he here ask—will the profession of Pennsylvania take this matter in hand? We opine, that the anatomical wants of Philadelphia alone will equal those of any single State in the Union; yet we hear of no effort in progress or contemplation, by which she is to be protected in her attempts to keep up a supply. It may be argued against the agitation of this question, that there is an abundance of material to be had, without a resort to legislative enactment; that well enough should be let alone. But, what security have the physicians and medical schools of this State, more than those of any other, that this supply will continue, without guarding its sources?

We have this winter witnessed the beginning of evils connected with the present system, which can be put a stop to in the future only by the strong arm of the law. What legal right have we to forbid the exportation of subjects to New York, or Baltimore, or wherever they will command a higher price than we are accustomed to pay? And, indeed, with what justice can we so much as complain, seeing that we take no proper steps to prevent it? Are not the miserable creatures engaged in this traffic upon an equal footing with ourselves, the consumers, so far as *right* is concerned? And every one will admit that their *might* is even superior to ours.

We earnestly hope that it will not be long, ere every State in the Union will do for its medical men and medical colleges, in reference to this matter, what is so clearly demanded by considerations of justice and humanity. But, to accomplish the ends desired, the physicians of the State, and, more especially, the professors of the medical colleges of Philadelphia, must bestir themselves; for, certainly, if they do not look after their own interests, it is not likely that the Legislature will do it for them.

---

One of the most marked and valuable results obtained by modern statistics in scientific matters, is seen in the establishment of certain general laws in regard to the currents of the air and ocean; and Prof. Maury has thus so completely annihilated the force of our common simile "instable as the wind," that unless we adopt the familiar negro phrase in reference to the uncertainty of the white man, sacrificing elegance of diction to the rude assertion of a fact, we are at a loss where to find a like method of expression, the significance of which is comparable to the one thus scientifically destroyed. But even here, and by the same square rule and dividers, our resources of language are again at fault, for M. Quetelet has clearly proved that the perversities, vices, and criminal acts of mankind are subject to the influence of general laws. In communities, man commits the same number of murders each year, and with the same weapons. We can now enumerate, beforehand, making exceptions for epidemics of moral delinquency, how many individuals will imbrue their hands in the blood of their kind; how many will commit forgery; how many will poison themselves or others, with very nearly the same accuracy with which the political economist can tell how many births and deaths will take place.

---

From a communication made by Dr. R. C. Wood, of the Army Bureau at Washington, to Robert Thompson, of Columbus, Ohio, and published in the last number of the Western Lancet, we learn that the total number of applicants for admission into the medical corps of the army, for the fifteen years ending with 1853, was 698. Of this number only 394 reported themselves to the Board of Examiners; 132 withdrew after a partial examination, or were decided to be physically disqualified; 284 were examined, and 114 approved. It appears, therefore, of the number of candidates examined, nearly 44 per cent. are approved, or 1 in every 2 or 3. The number who pass a satisfactory examination out of the whole number reported is nearly 29 per cent.

---

Drs. L. A. Dugas and H. Rossignol have resigned the editorial supervision of the Southern Medical and Surgical Journal (Augusta, Ga.), and are succeeded by Drs. Henry F. and Robert Campbell, the former Professor of Surgical and Comparative Anatomy in the Medical College of Georgia, and the latter Demonstrator of Anatomy in the same institution. Under its new auspices, the "Journal" presents an improved appearance; and if the first two numbers which we have received are to be taken as samples of what the future will be, we shall have abundant occasion to congratulate the profession of the Southeast upon having a medical periodical worthy of their support.

---

Dr. J. N. Douglas is hereafter to be associated with Dr. E. H. Parker in editing the American Medical Monthly, in New York; they are also proprietors of the work, which commenced its seventh volume with the beginning of the year.

---

The Western Lancet comes to us in an enlarged form and a new dress becoming the excellent matter with which it is filled. Prof. Blackman, of the Medical College of Ohio, is now the sole editor and proprietor.

---

Gustav. C. E. Weber, M.D., of New York, has been appointed Professor of Surgery in the Cleveland Medical College in place of Prof. Ackley, resigned. Dr. Weber is the son of the eminent German anatomist of that name, whose life-sized plates, illustrating the anatomy of the human body, are to be found in nearly every medical school in this country.

---

Dr. Brown Séquard, having returned from his visit to Charleston, where he has been delivering a course of lectures on the structure and functions of the spinal cord and nerves, will remain some time in this city. We learn that he has not yet determined upon a permanent residence.

---

Dr. J. Aitken Meigs has been appointed Professor of the Institutes in the Philadelphia College of Medicine, Dr. H. Hartshorne having been transferred to the Chair of Practice. Dr. W. Hembel Taggart has also been elected Professor of Materia Medica in the same College, in place of Dr. Tyson, resigned.

---

At a meeting of the Philadelphia County Medical Society, held on the 21st of January, the following officers were elected for the ensuing year:

*President.*—Dr. Gouverneur Emerson.

*Vice Presidents.*—Dr. John Bell, Dr. Thomas H. Yardley.

*Recording Secretary.*—Dr. J. Aitken Meigs.

*Assistant Secretary.*—Dr. T. Hewson Bache.

*Corresponding Secretary.*—Dr. Francis West.

*Treasurer.*—Dr. R. P. Thomas.

*Censors.*—Dr. John B. Biddle, Dr. D. F. Condie, Dr. R. La Roche, Dr. Samuel Lewis, and Dr. Lewis Rodman.

---

Destruction of the University of Louisville.—Most of our readers have, doubtless, ere this, heard of the destruction by fire of the noble edifice of the Medical Department of the University of Louisville. It occurred on the 30th of December last, at 8 o'clock in the morning, in consequence, as was supposed, of a flaw in the stove-pipe in the chemical laboratory, from which it rapidly spread to the museum, situated immediately above, and thence to the other apartments, the whole building being soon wrapped in flames. Great efforts were made to save its contents; but, with the exception of about two-thirds of the library and of the chemical apparatus, everything was destroyed. The total loss is estimated at $40,000, of which $32,000 only was covered by insurance. The edifice was one of the largest and most commodious of its kind in America, having three spacious and elegant lecture-rooms, a large museum, library, and dissecting-room, and private apartments for each member of the faculty; in short, every possible accommodation and convenience that such a building can have. The museum contained the best collection of skulls in plaster of Paris in the country, numerous dry and wet preparations, and a suite of Thibert's models of morbid structure, together with a valuable collection of fossils, which were totally destroyed.

Included in this destruction, was a portion of the private library of the senior editor of this journal, which had been sent there only a few days before his removal from Kentucky, for safe keeping. It comprised at least 2000 volumes, embracing a large collection of the older writers on surgery and anatomy, a number of series of medical journals, many volumes of medical tracts, and one of the most extensive and valuable collection of monographs on the diseases of the genito-urinary organs, in the world. Of the latter, many were from the library of the late Dr. Crosse, the eminent surgeon of Norwich, England. Not a volume of this collection was saved, nor was there any insurance upon it.

It is amazing how such a destructive fire could have originated; the occurrence implies great carelessness on the part of those who had the superintendence of the building, and should serve as a warning to the guardians of similar institutions. The day for stoves in public buildings has passed by, and those who continue to use them, in the face of the many modern improvements for heating houses, should be deprived of the benefit of insurance. That the whole edifice of the University of Louisville should have been destroyed in open daylight, will not surprise any one, when he is informed that Louisville has no public waterworks; the only city, probably, of its size in the world, that is in this predicament.

Notwithstanding the loss of their building, museum, apparatus, and library, the faculty are engaged in delivering their courses of lectures, and it is understood that they will take early measures for the erection and furnishing of a new edifice. What adds to their misfortune is, that the class has been unusually small this session, numbering less than at any period since the third year of the existence of the school.

---

THE NEW ANÆSTHETIC.—Our London exchanges contain accounts of experiments by Dr. John Snow, on the use of the vapor of amylene, by which it appears that this substance possesses anæsthetic properties not inferior to chloroform and ether. Dr. Snow first tried the effect of this, hitherto but little known remedy, upon some of the inferior animals, and witnessing no ill effects, he took the vapor himself with a like result. Since then, he has administered it to quite a number of patients in the London hospitals, with very good results. Mr. Fergusson, Mr. Bowman, and others, have allowed it to be employed in some of their operations, and express themselves as satisfied with its good effect in causing an entire prevention of pain, and an apparently pleasant sleep. Amylene is made by distilling fusel oil with chloride of zinc; its composition is 10 atoms of hydrogen, and 10 of carbon; its specific gravity is somewhere in the neighborhood of ·700; and its boiling-point 102° Fahr. It was first discovered by Cahours, about fifteen years ago, but Dr. Snow has the entire credit of demonstrating its anæsthetic properties. Its relative advantages and disadvantages, as stated by Dr. Snow in the Times and Gazette, are as follows: "In regard to its odor, it is more objectionable than chloroform, but much less so than sulphuric ether. In respect to its pungency, it has a great advantage over both ether and chloroform, being much less pungent than either of them. Thus, whilst the patient, especially if a female, often complains of a choking feeling and want of breath in commencing to inhale chloroform, and two or three minutes are lost before the vapor can be inhaled in any useful quantity, she can begin to inhale the amylene in full strength within half a minute from commencing, and the operation may generally be begun within three minutes. In the amount which suffices to induce insensibility, it is intermediate between chloroform and ether, chloroform having the advantage. Amylene has an advantage in preventing pain, with a less profound stupor than that occasioned by the other agents, and in the ready waking and recovery of the patient it has an advantage over chloroform, and a still greater advantage over ether. Its probable safety I have spoken of; and the greatest advantage of all, if it should continue to be met with in all cases, is the absence of sickness from its use. The almost entire absence of struggling and rigidity may also be mentioned as an advantage of amylene over ether and chloroform."

---

The election of Dr. Mayo to the presidency of the London College of Physicians, as successor to the late Dr. Paris, seems to give great dissatisfaction to the English journalists. It appears that the Elects are but seven in number, namely: Drs. Alderson, John Bright, Mayo, Francis Hawkins, Southey, and Turner, nearly all of whom are very old and decrepid, one being blind, another a cripple, another a mesmerist; so that the list of efficient men was very limited, and Dr. Mayo was not in this list. The presidency of the College is looked upon as the highest honor to which a medical man in Great Britain can attain, and in point of general influence, and more especially influence upon government officials and politicians, it is unlike anything of the sort in this country. Hence the great necessity for an active, energetic, shrewd man to fill the place, and hence too, the great disappointment produced by the recent election.

---

A British merchant vessel, called the Duke of Portland, engaged in carrying Chinese from Hong Kong to the Havana, lately lost 132 of these poor creatures on a single trip. The Times and Gazette may well ask, "What are the horrors of the middle passage in slavers to this? The causes of death were entered in the log-book as 'fever' and 'exhaustion.' Probably 'scurvy' and 'starvation' would have been nearer the truth."

---

M. Humboldt, in his "Personal Narrative," states that "in the thirteenth century the habit of eating human flesh pervaded all classes of society. Extraordinary snares were spread for physicians in particular. They were called to attend persons who feigned to be sick, but who were only hungry, and it was not in order to be consulted but devoured."

---

Mr. Alfred J. Wood, Surgeon to the Gloucester Infirmary, reports to one of the London journals, what he claims as a new suture for the cure of hare-lip, and refers to a case published in the Medical Gazette, so long ago as Sept. 24, 1841, as the first in which he had employed it. He states that since that time he has used it in all cases in which hare-lip needles and the twisted suture are generally applied, and with uniformly successful results. Without the necessary illustrations, we cannot give our readers a very clear idea of the nature of this suture, but may state that it is not unlike the ordinary shotted suture; only instead of metal ligatures, Mr. Wood uses silk, and instead of the shot, a similar number of little silver discs perforated at the centre with a bar across the opening, upon which to tie the threads. A large straight needle is armed with a double ligature, the two ends of which are slipped through one of the discs and tied over the bar; the needle is then carried entirely across the wound, the thread drawn so as to bring the disc to press upon the lip, when the needle is cut off, and the two ends carried through another similar disc in the same manner as the first. Upon slipping the second disc down to its place, and drawing upon the ligature, the opposite sides of the wound are brought in contact, when the two threads are tied across the bar. It is seldom that more than two such sutures are required in cases of hare-lip.

We need hardly call attention to the fact that this suture, although bearing a very close resemblance, is not a modification of the clamp-suture, having been invented long before the latter was thought of. Its applicability to all wounds where deep sutures are required, and where the quill-suture is customarily employed, as in the operation for lacerated perineum, is pointed out by Mr. Wood.

---

Dr. Livingston, who lately returned to Great Britain, from an absence of nearly seventeen years in the wilds of South Africa, is undergoing the ordeal of a degree of public attention, quite unusual with our phlegmatic neighbors. But he well deserves it and tenfold more. As an example of a persevering and self-sacrificing man of science and a zealous missionary, he has probably no equal in the ranks of the Christian Church, or the medical profession. In addition to all, his modesty in positively refusing to publish to the world the details of the dangers he has encountered, is equally rare, and although carried to an unnecessary extreme, forms an agreeable contrast to the romantic accounts of some professedly scientific travellers, whom we might name. In one of his public addresses, he, however, mentioned incidentally that for *sixteen years* he had not an opportunity of conversing in his native language, a circumstance which is of itself significant to our minds of positive suffering seldom endured, even within the walls of a state prison. His contributions to scientific knowledge have not yet been given to the public, but doubtless will be ere long, when we look for some most interesting additions to our present slender information, concerning the diseases of the countries through which he travelled. In his address before the London Missionary Society, he stated that some of the interior districts of Africa are perfect sanatoria, and among the pure negro family, many diseases common in Europe are almost unknown. Small-pox and measles had not been known for twenty years, and consumption, scrofula, cancer, and hydrophobia are seldom heard of.

---

Now that the smoke of the contest, conducted, on the one hand, by Mr. Fergusson, and on the other by the equally distinguished Mr. Syme, in regard to the manner in which the late Mr. Liston was in the habit of holding the knife in the operation of lithotomy, has passed away, it turns out as we predicted, that the Scotch professor has gained the day, and the victorious party are having a gay time over the defeat of the Queen's surgeon. The last and decisive charge was made by Mr. Pirrie, whose array of facts in proof that Mr. Liston held the scalpel in the underhanded position, as represented in the various wood-cuts which appear in the pages of his own, Mr. Pirrie's, Mr. Miller's, and Mr. Druitt's books on surgery, is perfectly overwhelming.

But hardly is this *great* battle of the knives decided before another equally honorable to the distinguished parties involved is begun. Mr. Ramsbotham, who, however, notwithstanding his high position in the obstetric world and the adaptative nature of his calling to the annually recurring professional wants of her Majesty, has not, we believe, the entrée of the royal chamber, charges publicly upon Mr. Churchill, a brother accoucheur, a theft committed some twelve or fifteen years ago. The articles said to be stolen are certain wood-cut illustrations, which, without leave or license, or even an acknowledgment, Mr. Churchill was wicked enough to copy from Mr. Ramsbotham's book on midwifery, published about that time. The war has just begun, and how it will end the world will no doubt in due time be informed. In the meantime, speculation is rife, and the intensest interest is felt to know the result.

---

WHEN IS A MEDICAL TEACHER OR PRACTITIONER SUPERANNUATED?—In the organization of one of our western medical schools some years ago, the board of trustees of the institution declared, by the enactment of a statute, that when a professor has attained the age of sixty-five years he is no longer qualified to teach, but must consider himself superannuated, in spite of whatever testimony he may have to the contrary. In Paris, a law prevails in regard to hospital physicians and surgeons, similar in its bearing, but even still more arbitrary and unjust, since its limit is five years less. Under this regulation MM. Paul Dubois, Cruveilhier, Bonneau, and Hervez de Chégoin, having reached the fatal sixtieth year, retired from their respective posts at the beginning of the present year. M. Paul Dubois continues, however, as Professor of Clinical Midwifery to the Faculty at the Hôpital des Cliniques; it is at the Maternité that he will have a successor.

---

ARABIAN SURGERY IN THE FIFTH CENTURY.—The following description by Hacam-Addimechky of the application of pressure and water-dressing for the cure of a wounded artery, will be read not without some degree of interest. It is contained in a History of Physicians, by Aby Ossaibi'ah, who was born in Damascus at the beginning of the thirteenth century.

"I was," said Ica, "in the company of my father in the city of Damascus; we were passing by the shop of a kind of barber or surgeon-cupper, opposite which a crowd was collected. One of the number having recognized us, said: 'Here is Hacam, the physician, and his son Ica; make room.' The people gradually withdrew, and we then saw a man who had been bled by the barber in the basilic vein. The orifice was large—the basilic was over the artery; the operator did not know how to isolate the vein, and he wounded the artery. The surgeon-barber did not know any means of arresting the flow of blood. We tried compresses, spider's web, fine hair, but without success. My father asked me if I could propose any other plan, and I replied in the negative. He then told me to bring a pistachio nut, which he broke into two equal parts, and, throwing away the fruit, took one half of the shell and placed it on the opening made by the bleeding. He then cut a roller of thick linen, with which he enveloped the wound over the half of the pistachio nut, drawing the bandage so tight as to make the man cry out for help. Having thus inclosed the arm, he secured well the ligatures, and directed the man to be taken to the river Barada. My father then made him put his arm into the river—while resting on a kind of bed on the bank, and left him to sleep there. He also made him swallow some yolks of eggs half-boiled, and left him under the care of one of his pupils. My father enjoined the latter not to permit the patient to withdraw his arm from the water, except at the hour of prayer, unless there was reason to fear that death might ensue from the extreme cold. In this case the man was to be permitted to take his arm out of the water for a few moments, and then immediately to immerse it again. This lasted till the evening. My father then had the wounded man taken home, and he forbade him to uncover the wound from the bleeding, or to take off the bandage until five days had elapsed. The man obeyed, but my father went to see him on the third day, and saw that the arm was very much swelled, as was also the forearm. He then loosed the ligature; and told the patient "the swelling is a less serious thing than death." On the fifth day, my father removed the bandage, and we found that the shell of the pistachio was adherent to the flesh. My father told the individual, it is by this shell that you have been saved from the tomb; if you take it away before it separates. and falls off of itself, you will bring about your own death. The shell fell off on the seventh day, and in its place there was dry blood, and a clot of the figure of the pistachio nut. My father told this man not to touch the clot, nor to scratch around its border, nor to attempt to break it with his fingers. The blood came away by degrees, and the place of the bleeding was only visible after the lapse of forty days. This man was completely cured.

---

GROSS INJUSTICE TO AN EMINENT PHYSICIAN.—We read, in the *Arch. Gen.* for December last, a condensed notice of the arbitrary treatment to which Dr. COINDET, the long and well-known physician of Geneva, has been subjected by the Council of State of that city, at the instance of a Dr. Duchosal, President of the Department of Justice and Police. A full narrative of the whole transaction has been published by the Medical Society of Geneva. The members of the Faculty of Medicine of that city have, almost by unanimous vote, sent an address of condolence to Dr. Coindet, in which the conduct of the government and its chosen experts are emphatically blamed.

It seems that Dr. Coindet, who has been at the head of the Asylum for the Insane for the last twenty-five years, was consulted, not long since, respecting the condition of a young girl, who he soon discovered was laboring under periodical insanity. In place, however, of giving, as he had the right to do, a simple certificate which would authorize her being placed in a *maison de santé*, or private establishment for the sick, he took the precaution, as the case was one of insanity, the diagnosis of which was difficult, to call on Dr. Duchosal, a counsellor of state and a public officer, whose functions have just been named, and gave all desirable explanations respecting the mental condition of the young girl. In consequence of this information, Duchosal signed the order for her admission into the Asylum for the Insane, and had her removed to the institution by one of his agents.

A fortnight after this, Duchosal appointed two experts who did not belong to the commission legally intrusted with this duty, but who were charged with that of examining anew the actual state of mind of this young girl. These two persons and two others, political friends of the counsellor of state, acting without the knowledge of Dr. Coindet, drew the notable conclusion, after a very imperfect report, that at the *moment of their examination* the young girl exhibited no evidence of insanity. On the strength of this report, the Council of State, without waiting for any other document, but wishing, while it accomplished its purpose, to shield itself from the odium of an unjust dismissal from his office, accused Dr. Coindet, in writing, of the arbitrary detention of a young girl, who was a minor; and after having thus calumniated him, sent him a derisory invitation to resign his post in the Asylum for the Insane. On the refusal, stated on strong grounds, by Dr. Coindet, the Council dismissed him from office; and reiterated, at the same time, the accusation against him of illegal sequestration.

This brutal dismissal (as it is called by the French Journalist), without investigation or discussion, produced a deep sensation in the Medical Faculty of Geneva, which body hastened to address Dr. Coindet in the manner already stated. But their letter was made the signal for a fresh display of arbitrary power, in dismissals from office, as unjust as that of Dr. Coindet, of most estimable men, known to all for their scientific attainments and honorable deportment; but who were accused or suspected of having signed the letter of protest.

